# YouTube Videos on Osteochondral Allograft Transplantation Lack Accuracy and Quality

**DOI:** 10.1016/j.asmr.2025.101156

**Published:** 2025-04-25

**Authors:** Sebastian Schmidt, Alexander Bumberger, Luis Navas, Chilan B.G. Leite, Domenico Franco, Ali Darwich, Christian Lattermann

**Affiliations:** a Department of Orthopedic Surgery, Brigham and Women's Hospital, Harvard Medical School, Boston, Massachusetts, U.S.A.; b Department of Orthopaedic and Trauma Surgery, University Medical Centre Mannheim, Medical Faculty Mannheim, University of Heidelberg, Mannheim, Germany; c Department of Orthopedics and Trauma Surgery, University Hospital Vienna, Medical University of Vienna, Vienna, Austria; d ARCUS Kliniken, Department of Sports Medicine, Pforzheim, Germany; e Operative Research Unit of Orthopaedic and Trauma Surgery, Fondazione Policlinico Universitario Campus Bio-Medico, Rome, Italy

## Abstract

**Purpose:**

To evaluate the accuracy and informational quality of YouTube videos related to osteochondral allograft (OCA) transplantation as a potentially valuable educational resource for patients and health care professionals.

**Methods:**

A systematic analysis of YouTube videos retrieved through a predefined search strategy using the key words “osteochondral allograft” was performed. Videos were categorized by content sources, such as health care professionals with and without commercial bias, individuals, or personal testimonials. The video’s duration, the publication date, and number of likes and views were recorded. To evaluate the accuracy, reliability and quality of video content, each video was assessed using the *Journal of the American Medical Association* (JAMA) benchmark criteria, Global Quality Score (GQS), DISCERN, and a newly developed Osteochondral Allograft Quality (OCA-QAL) score, designed specifically for this procedure.

**Results:**

In total, 80 YouTube videos were included. Overall, the quality of OCA-related YouTube videos was low, with mean scores of 2.16 (JAMA), 2.28 (GQS), 32.58 (DISCERN), and 5.71 (OCA-QAL). Only one video was rated as “excellent” on OCA-QAL, and none achieved full points on JAMA or GQS. Video categories included educational content with (27.5%) or without (51.3%) commercial bias for health care professionals, content for nonhealth care individuals (13.8%), and testimonials (7.5%). Strong positive correlations emerged between OCA-QAL, GQS, and DISCERN scores, whereas views and likes did not predict quality.

**Conclusions:**

YouTube videos on OCA transplantation generally do not meet the quality standards like peer-reviewed validation necessary for reliable patient education. Given the low quality of available content, health care providers should be cautious in recommending YouTube as a resource for OCA transplantation information and should guide patients to more rigorously reviewed resources.

**Clinical Relevance:**

As cartilage procedures like OCA transplantation become more common, surgeons and patients lack reliable online resources. This study underscores the need for improved digital health content to ensure accurate and trustworthy patient education.

Articular cartilage defects can result from multiple factors, including trauma, degeneration, avascular necrosis, osteochondritis dissecans, or osteoarthritis.^1^ Studies have indicated that cartilage defects may occur in up to 66% of the cases in various joints, leading to symptoms such as swelling, pain, and restricted mobility.^2^, ^3^, ^4^ Over time, focal cartilage defects can progress to diffuse cartilage degradation, increased pain, reduced joint stability, and the potential development of diffuse osteoarthritis, ultimately resulting in the loss of joint function.^1^^,^^2^^,^^5^^,^^6^

According to the characteristics of the cartilage injury, different reparative and restorative treatments have been performed, showing favorable outcomes.^1^^,^^3^^,^^6^ For large defects >4 cm^2^ or if the underlying bone has to be restored in addition to the cartilage, osteochondral allografts show improved function, satisfaction and pain relief.^6^, ^7^, ^8^ Osteochondral allograft (OCA) transplantation is a restorative procedure used to address symptomatic focal cartilage defects or osteochondral lesions by transplanting viable mature hyaline cartilage along with subchondral bone into the affected area.^9^, ^10^, ^11^ Several studies show satisfactory long-term clinical results after OCA transplantation in different joints, with allograft survival rates at 10 to 25 years postoperatively between 59% and 91%.^12^^,^^13^ The widespread use of OCA transplantation has made it one of the most frequently performed cartilage repair procedures in the United States.^11^

In recent years, online searches for information on orthopaedic conditions and procedures increased substantially, as patients are becoming more independent and increasingly conducting their own research.^14^ Among the various online video platforms, YouTube (Google, San Bruno, CA) is a key source on which patients rely when seeking information about different surgical procedures as the result of its vast reach and accessibility. Previous studies have assessed the quality of content on YouTube regarding anterior cruciate ligament reconstruction, patellofemoral instability, and rotator cuff pathology, with the authors finding that the information available is generally of low quality.^15^, ^16^, ^17^, ^18^ However, these videos have been found to significantly influence patients' decisions on whether to undergo a particular procedure.^19^ At the same time, surgeons also use YouTube as a resource for continuing education and learning new surgical techniques.^20^

OCA transplantation was chosen for this study because its increasing prevalence and complexity make it a critical case for evaluating YouTube’s reliability as an educational tool, especially given the limited attention to cartilage procedures in previous research. The purpose of this study was to evaluate the accuracy and informational quality of YouTube videos related to OCA transplantation as a potentially valuable educational resource for patients and healthcare professionals. The hypothesis was that the video content on this platform would not offer sufficient or reliable information and should therefore be recommended to patients with caution.

## Methods

This study did not require approval from the institutional review board. It focused on evaluating YouTube videos related to OCA transplantation. The data that support the findings of this study are not openly available due to reasons of sensitivity but are available from the corresponding author upon reasonable request. The YouTube search was conducted using the specific key words “osteochondral allograft” AND (“transplantation” OR “patient” OR “cartilage” OR “sport”) and the number of videos retrieved for each key word was recorded (search conducted on August 20, 2024). Two experienced surgeons specializing in knee surgery (S.S. and C.B.G.L.) independently conducted and validated the search. The anonymous Web browsing The Onion Router (TOR) software (The TOR Project, Seattle, WA) was used to avoid biased search results as the result of topographic factors and browsing behavior as previously described.^16^^,^^17^ Results were ordered by relevance as the result of YouTube’s default settings. According to studies, users do not access videos beyond the third page.^15^^,^^21^ Hence, only the first 100 results per search term were included.^21^ Videos that were off-topic, non-English, without sound, duplicates, short reels, podcasts or those with poor pronunciation were excluded from the analysis.

The study recorded (1) uniform resource locator (URL), (2) video title, (3) number of total views, (4) video category, (5) duration of video in minutes, (6) date of publication, (7) days since upload, (8) number of likes, (9) number of dislikes, (10) like ratio, (11) Video Power Index (VPI), and (12) view ratio (views per day). The like ratio is calculated using the formula: (likes × 100) / (likes + dislikes). The VPI measure is used to a video's popularity on the basis of its views and likes and has been used in previous research.^16^, ^17^, ^18^ It is computed as: (like ratio × view ratio) / 100. All videos included in the study were classified into the following categories: educational (health care professionals) with/without commercial influence, educational (nonhealth care professionals) with/without commercial influence or personal testimony. Discrepancies were clarified by consensus discussion by the authors. To evaluate the video quality, reliability and validity multiple scoring systems were used.

To assess the content quality, the Global Quality Score (GQS) was chosen.^22^ The GQS includes 5 criteria, each earning 1 point if met, with a maximum score of 5 points indicating high educational value, providing a quick, broad quality overview. Moreover, the *Journal of the American Medical Association* (JAMA) benchmark criteria were used to evaluate the accuracy and reliability of the videos. This score is summed on the basis of 4 specific criteria, allowing for an objective assessment. Each criterion earns one point, with a maximum possible score of 4 points, where a greater score indicates greater reliability and accuracy.^23^ It excels as a strength in ensuring source credibility. Another commonly used scoring system to assess reliability and quality is the DISCERN score, which consists of 16 questions and an overall rating on the basis of these questions.^15^^,^^17^^,^^24^ Each question is scored from 1 to 5 points, with a total possible score ranging from 16 to 80 points. A greater score reflects better content quality. Its strength is in evaluating treatment comprehensiveness.

To assess the educational quality of online videos, an accepted approach in the orthopaedic field is to use nonvalidated and novel assessment tools.^15^^,^^17^^,^^25^ A new 25-item scoring system (Osteochondral Allograft Quality Score, OCA-QAL Score) was developed on the basis of elements of anatomy, diagnosis, treatment options, and rehabilitation (Table 1).^25^, ^26^, ^27^, ^28^ Quality was assessed by one point per criteria contained in the video, with a maximum score of 24 points, offering OCA-specific detail. Scores were adapted for different joints (knee, ankle, and shoulder). On the basis of the overall score, the quality of videos was considered excellent (21-24 points), good (16-20 points), moderate (11-15 points), poor (5-10 points), or very poor (<5 points). The assessment was performed independently by 2 experienced surgeons specializing in knee surgery (S.S. and C.B.G.L.) with an interval of 4 to 6 weeks to avoid bias.

**Table 1:** Scoring Items of Osteochondral Allograft Quality Score (Maximum 24 Points)

One point each:
Describes function of articular cartilage
Differentiates articular cartilage from meniscus cartilage
Describes causes of cartilage damage
Mentions poor healing potential of articular cartilage
Differentiates cartilage defect from generalized arthritis
Describes symptoms
Mentions cartilage defect can progress to arthritis
Describes use of magnetic resonance imaging to evaluate cartilage defects
Defines candidates for surgery (i.e., young adults with single lesion)
Defines noncandidates for surgery (i.e., older patients, multiple defects, generalized arthritis)
Mentions surgery can be completed arthroscopically or open
Describes other problems (i.e., ligament tears, malalignment) that may be addressed at surgery
Mentions other cartilage therapies (i.e., ACI, AMIC)
Mentions matching criteria (on the basis of ABO blood type, no anti-immunogenic drugs are required)
Explains why other therapies don't apply
Describes complications
Mentions restricted weight-bearing after surgery
Mentions need for postoperative physical therapy
Mentions postoperative continuous passive motion
Describes timeline of return to function
Mentions factors with less-favorable outcome
Mentions implant survival
Mentions outcome
Revision surgery (arthroplasty, Re-OCA)
Total

ACI, autologous chondrocyte implantation; AMIC, autologous matrix-induced chondrogenesis; OCA, osteochondral allograft.

### Statistical Analysis

Categorical data are presented as absolute and relative frequencies, whereas continuous data are expressed as mean values with their standard deviations. Exploratory data analysis was performed to assess variable distributions. The Kolmogorov-Smirnov test was applied to determine if the analyzed variables followed a normal distribution. Because not all variables met the normal distribution criteria, the Kruskal-Wallis test with Dunn test correction was used for comparison.^29^ Correlations were classified according to the Altman criteria, with values considered poor (0.00-0.20), fair (0.21-0.40), moderate (0.41-0.60), good (0.61-0.80), or excellent (0.81-1.00). Multivariate linear regression was used to analyze the impact of specific video characteristics on the quality and reliability of the assessed videos. Intraobserver and interobserver reliability were assessed using intraclass correlation coefficients, where values greater than 0.75 were considered to indicate good reliability. A significance level of *P* < .05 was applied to the results. Statistical analysis was performed by using R, version 4.4.1.

## Results

**Fig 1:**
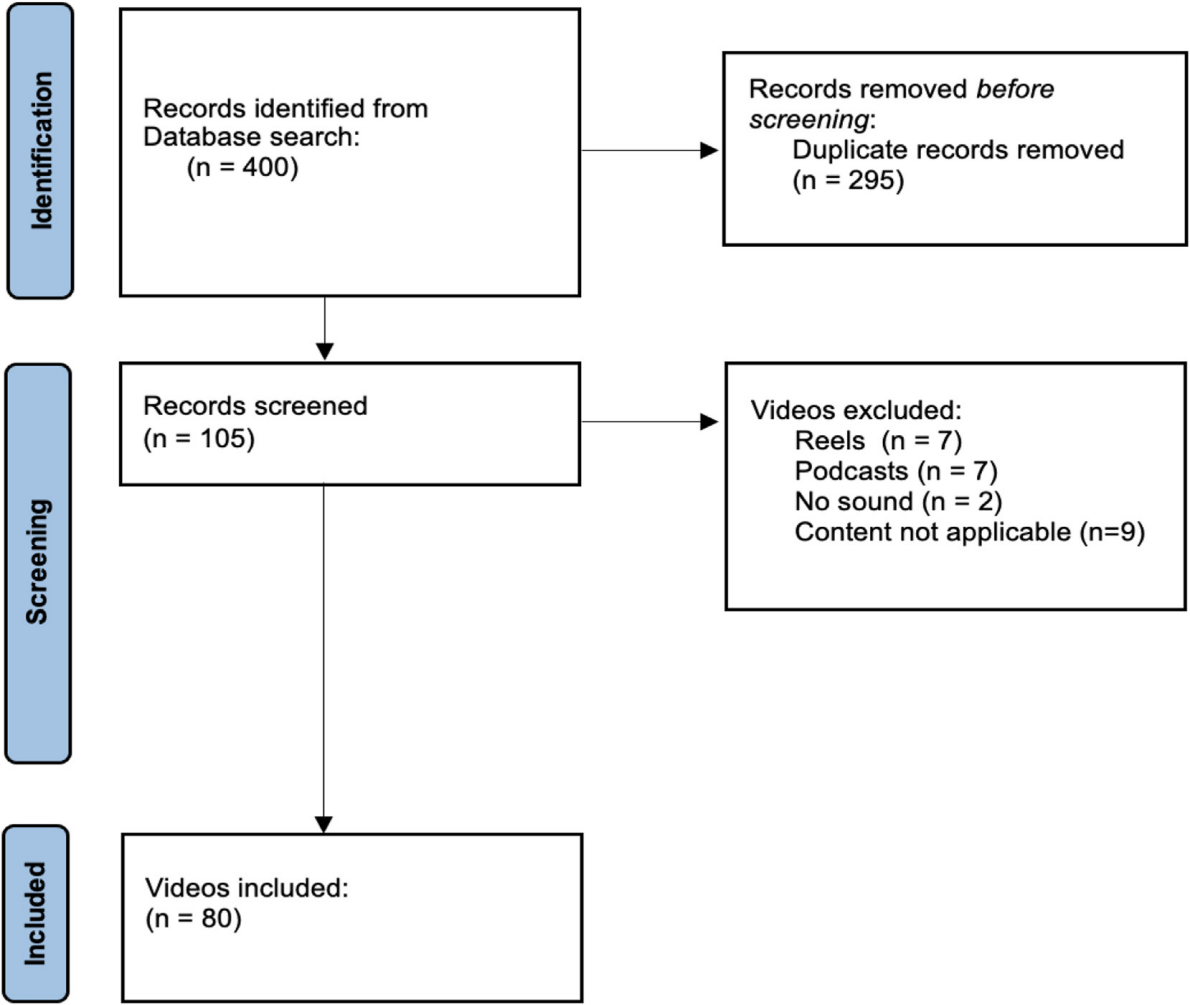
Flowchart of the video screening performed in this study.

A total of 80 videos were included in the analysis. A flowchart detailing the video selection process is provided in Figure 1. Of these, 22 (27.5%) were classified as educational for health care professionals with commercial content. In addition, 41 (51.3%) contained educational content for health care professionals without advertising content. Eleven (13.8%) videos had content for nonhealth care individuals without advertising content. Only 6 (7.5%) videos were testimonials. In terms of joint involvement, 65 (85%) videos were related to the knee joint, 6 (7.5%) to the shoulder and elbow, and 7 (8.8%) to the ankle or foot. The other video characteristics can be seen in Table 2.

**Table 2:** Characteristics of the Videos Included in the Study

	n (%)
Video category	
Educational health care with commercial	22 (27.5)
Educational health care without commercial	41 (51.3)
Educational nonhealth care without commercial	11 (13.8)
Testimonial	6 (7.5)
Joint	
Knee	68 (85)
Shoulder/elbow	6 (7.5)
Ankle/foot	7 (8.8)
	Mean ± SD (95% CI; range)
Video characteristics	
Video length, min	8.18 ± 11.31 (5.67-10.7; 0.87-66.25)
Days online	1,860 ± 892.52 (1,661-2,059; 129-4,970)
Total number of views	2733 ± 4,915.15 (1,639-3,827; 46-28,614)
Total number of likes	21.95 ± 37.51 (14-30; 0-192)
Total number of dislikes	0 (0; 0)
View ratio	2.1 ± 6.21 (1-3; 0.03-41.19)
Like ratio	80 ± 40 (71-89; 0-100)
Video Power Index (VPI)	2.08 ± 6.21 (1-3; 0-41.19)
Video scores	
JAMA	2.16 ± 0.65 (2.08 -2.23; 1-4)
GQS	2.28 ± 0.65 (2.21-2.35; 1-4)
DISCERN	32.58 ± 6.7 (31.84-33.31; 20-62)
OCA-QAL	5.71 ± 4.66 (5.19-6.22; 0-22)

CI, confidence interval; GQS, Global Quality Score; JAMA, *Journal of American Medical Association*; OCA-QAL, Osteochondral Allograft Quality Score; SD, standard deviation.

The average provided information quality, accuracy, and reliability were poor. The mean JAMA, GQS, DISCERN, and OCA-QAL scores were 2.16 ± 0.65 (2.08 −2.23; 1-4), 2.28 ± 0.65 (2.21-2.35; 1-4), 32.58 ± 6.7 (31.84-33.31; 20-62), and 5.71 ± 4.66 (5.19-6.22; 0-22), respectively. Not a single video fulfilled the criteria to obtain all points for either the JAMA benchmark criteria score or the GQS.

The intraobserver and interobserver reliability showed excellent intraclass correlation coefficients in all scores. Intraobserver reliability was: 0.93 (95% confidence interval [CI], 0.85-0.93), *P* < .001 for JAMA; 0.93 (95% CI, 0.86-0.95), *P* < .001 for GQS; 0.92 (95% CI, 0.88-0.95), *P* < .001 for DICERN and 0.99 (95% CI, 0.98-1), *P* < .001 for OCA-QAL. Interobserver reliability was 0.96 (95% CI, 0.95-0.97), *P* < .001 for JAMA; 0.96 (95% CI, 0.95-0.98), *P* < .001 for GQS; 0.97 (95% CI, 0.96-0.98), *P* < .001 for DISCERN and 1 (95% CI, 1), *P* < .001 for OCA-QAL.

**Fig 2:**
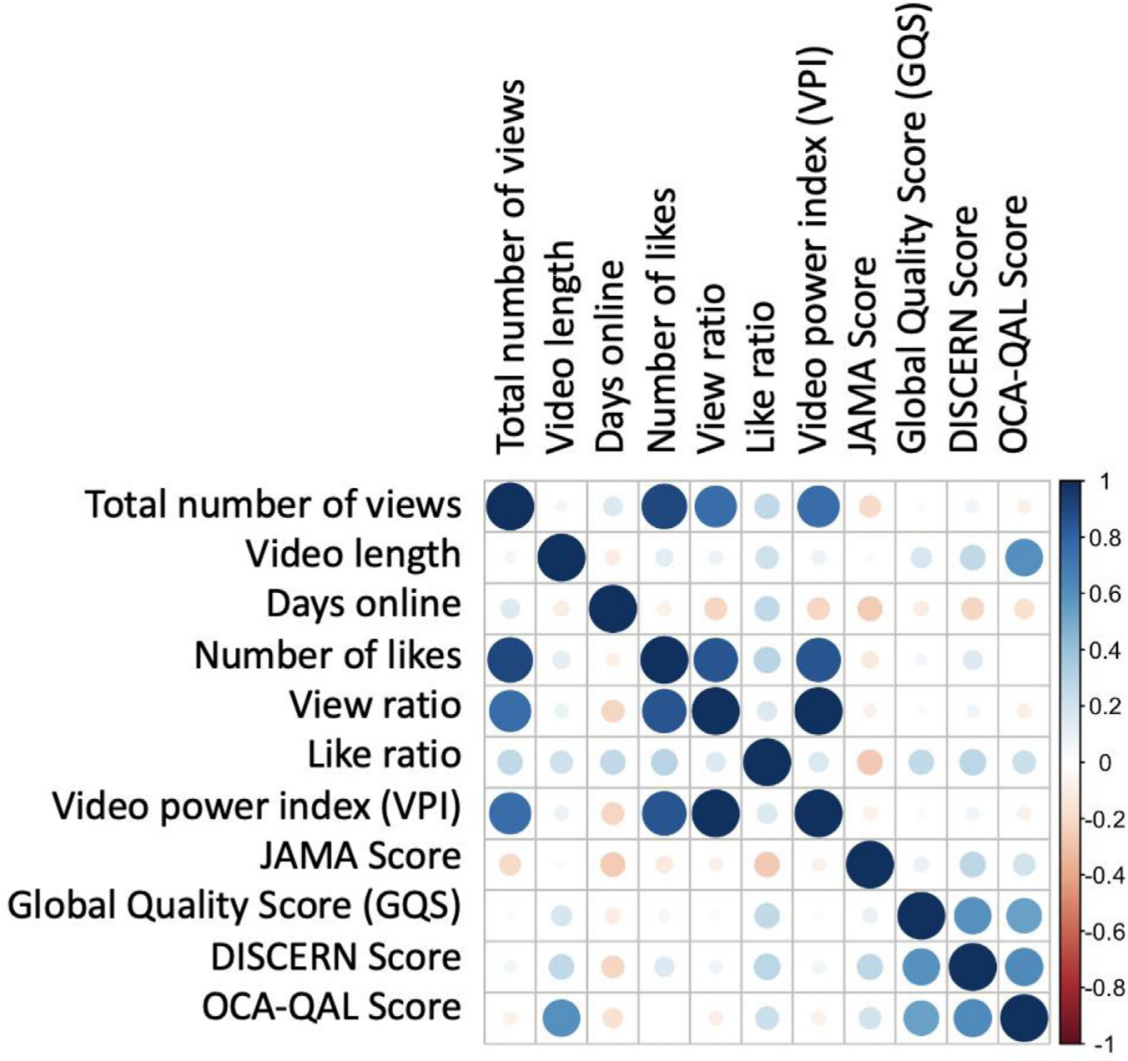
Correlation analysis between video characteristics. Color and size indicating correlation (blue: positive correlation; red: negative correlation). (JAMA, *Journal of American Medical Association*; OCA-QAL, Osteochondral Allograft Quality Score; GQS, Global Quality Score.)

The evaluation of the correlation between the scores and the numerical variables of the video characteristics revealed a strong correlation of the OCA-QAL with the GQS (0.5382; *P* < .001) and the DISCERN (0.6238; *P* < .001). The GQS also correlates strongly with the DISCERN (0.5938). Only the JAMA shows a weak correlation with the other scores (GQS: 0.097, *P* = .405; DISCERN: 0.2780, *P* = .013 and OCA-QAL: 0.2037, *P* = .072). Furthermore, a slightly negative correlation was found between the JAMA score and the total number of views (−0.1971) as well as days online (−0.2526). For the video characteristics, there was a high correlation between the total number of views and the total number of likes (0.9015) as well as a slight correlation with the video length (0.1282). All other correlations between the characteristics are shown in Figure 2. The OCA-QAL score also showed a high positive correlation with video length (0.6024), whereas the GQS and DISCERN only showed a weak positive correlation (0.1809, 0.2678).

**Fig 3:**
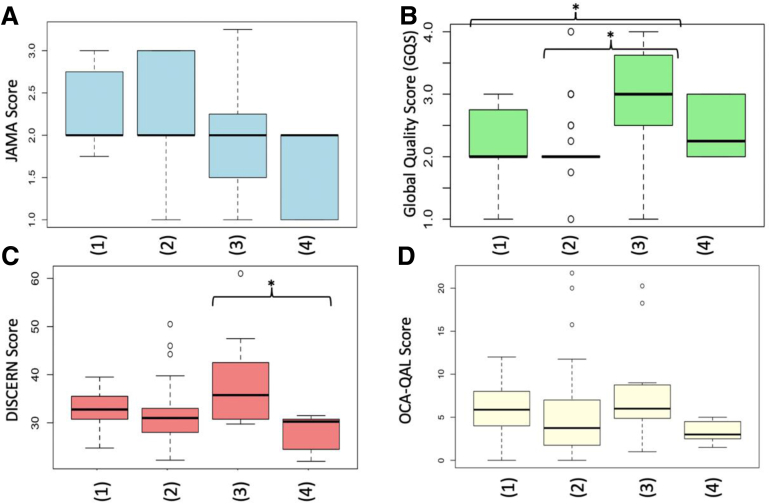
Scores of different video categories educational health care with commercial (1), educational health care without commercial (2), educational nonhealth care without commercial (3), and testimonial (4). Significance is indicated by ∗*P* < .05. (JAMA, *Journal of American Medical Association*; OCA-QAL, Osteochondral Allograft Quality Score; GQS, Global Quality Score.)

The evaluation of the results according to the video categories revealed significant differences between the individual groups for video length (*P* = .005, like ratio [*P* = .007], GQS [*P* < .001], and DISCERN [*P* = .015]), whereas there was no significant difference in JAMA or OCA-QAL score (Figure 3, *A* and *D*). The specific corrected pairwise differences showed significance between “educational health care with commercial” and “testimonial” for video length (6.72 ± 4.72 [95% CI, 4.62-8.81; range, 0.87-20.52]; 2.8 ± 0.94 [95% CI, 2.38-3.22; range, 1.42-3.92]; *P* = .043). For the like ratio, the comparison between “educational health care with commercial” and “educational health care without commercial” was significant (100 ± 0 [95% CI, 0; range, 100]; 65.85 ± 47.42 [95% CI, 50.88-80.82; range, 0-100; *P* = .008]). For the GQS, the difference between “educational health care with commercial” versus “educational non-health care without commercial” (2.18 ± 0.55 [95% CI, 1.94-2.43; range, 1-3; Figure 3, *B*]; 2.98 ± 0.86 [95% CI, 2.39-3.56; range, 1-4]; *P* = .006) and “educational health care without commercial” versus “educational nonhealth care without commercial” (2.12 ± 0.44 [95% CI, 1.98-2.2; range, 1-4]; 2.98 ± 0.86 [95% CI, 2.39-3.56; range, 1-4]; *P* = .0002) was significant. The comparison between “educational non health care without commercial” and “testimonial” was significant for the DISCERN score (38.34 ± 9.2 [95% CI, 32.16-44.52; range, 29-61]; 28.21 ± 3.62 [95% CI, 25.78-30.64; range, 22-32]; *P* = .036; Figure 3, *C*).

## Discussion

The key finding of this study is that the overall quality, accuracy, and reliability of information for OCA transplantation in YouTube videos are lacking. Patients and health care professionals are both increasingly using modern media platforms such as YouTube as a source of education and information.^25^^,^^30^, ^31^, ^32^ Other video platforms such as VuMedi, a network dedicated to medical training and information for doctors, are therefore not a relevant alternative. VuMedi is not freely accessible to the general population, even though it has been shown that the educational quality of surgical videos for health care professionals can be better than on YouTube under certain circumstances, whereby the potential benefits for patients as a source of information remain questionable.^32^ For this reason, VuMedi was excluded from the analysis in this study.

YouTube is not a peer-reviewed platform, and neither the content quality nor the references are verified. Although health care professionals can more effectively assess the information, inaccurate content could negatively impact patients’ expectations, patient-physician communication, and potentially even clinical outcomes.^17^^,^^19^^,^^30^ Patients, physicians, and physiotherapists should be aware that online videos cannot substitute an in-person consultation although YouTube remains highly accessible.^15^^,^^17^ Health care providers could specifically address this issue during consultations and guide patients to high-quality, peer-reviewed videos created by professional organizations ensuring access to reliable alternatives.

The quality of information on YouTube has already been evaluated in several medical fields.^17^^,^^22^^,^^31^ In the orthopaedic field, YouTube video content regarding a variety of diagnoses and procedures has been analyzed.^15^^,^^17^ The present findings align with previous research evaluating YouTube videos on various orthopedic conditions and knee joint surgeries.^16^, ^17^, ^18^^,^^33^

As shown in previous studies, most of the videos were produced by health care professionals, and this was a positive predictor of a greater GQS.^18^^,^^33^ In most cases, medical professionals should be addressed. These results were also found in this study, whereby there were no significant differences in the video groups due to the influence of commercials, except for the ratio. The authors suggest that a more positive like behavior or interaction with the videos for health care professionals is possibly triggered by the corresponding companies mentioned in the videos.

The JAMA, GQS, and DISCERN scores frequently used in the literature yielded poor results for the reliability and quality of the videos in this study, similar to those already described in the literature.^16^, ^17^, ^18^^,^^33^ The newly created OCA-QAL score was able to achieve excellent intraobserver and interobserver reliability in this study. Overall, it showed a good correlation with the GQS and DISCERN score. Only the JAMA score had a slight correlation, but this was also shown between JAMA and GQS as well as JAMA and DISCERN. This likely reflects its focus on credibility (authorship, references, disclosure, currency) rather than educational content quality, which GQS, DISCERN, and OCA-QAL prioritize. Of the videos included, only one achieved an excellent rating for the OCA-QAL and no video achieved excellent scores for JAMA and GQS, highlighting the difficulty encountered with non−peer-reviewed information, as well as the complexity of this topic, especially if information is provided by nonprofessionals.

Yüce et al.^18^ have shown in their work that at least 1 score correlates with the length of the videos. This was confirmed in this study. The OCA-QAL in particular showed a strong positive correlation. This fact makes it much more difficult for patients to identify high-quality videos, as there is no correlation between typical markers such as views or likes. Although the longest videos in this study were in the “educational health care with commercial” category, educational videos for nonhealth care professionals without commercials showed significantly better results in 2 of 4 scores.

Finally, the authors recommend improving the content quality of YouTube videos on cartilage repair procedures in the future to give patients the opportunity to inform themselves. In order to achieve a satisfactory quality of the videos, the scores mentioned in this paper should be considered, but at the same time, attention should also be paid to appropriate and understandable language for the patient. This was not taken into account in this study. Furthermore, an increased use of short videos, e.g., “reels,” has been observed among patients and medical professionals in recent years.^16^^,^^30^ They can be viewed by users without the need to actively search for them, which implies that they follow a different probability distribution regarding the likelihood of receiving Likes or views compared to classic content that requires direct searching. The effects of this condensed form of information on the quality should be the subject of future research in this area. Based on the strong correlation between video length and OCA-QAL values (r = 0.6024) in this study, longer videos better convey necessary depth, which leads to the risk that short videos risk oversimplification or misinformation.

### Limitations

This study has limitations. First, only English-language videos were included, which potentially limits generalizability. The scores used have been widely reported in studies but are ultimately not validated. Furthermore, this study is only a snapshot of the currently available information on YouTube and does not accurately show the relative fluidity of available information.

## Conclusions

YouTube videos on OCA transplantation generally do not meet the quality standards like peer-reviewed validation necessary for reliable patient education. Given the low quality of available content, health care providers should be cautious in recommending YouTube as a resource for information on OCA transplantation and should guide patients to more rigorously reviewed resources.

## Declaration of generative AI and AI-assisted technologies in the writing process

During the preparation of this work the authors used ChatGPT4o (OpenAI) in order to improve language. After using this tool/service, the authors reviewed and edited the content as needed and take full responsibility for the content of the publication.

## Disclosures

All authors (S.S., A.B., L.N., C.B.G.L., D.F., A.D., C.L.) declare that they have no known competing financial interests or personal relationships that could have appeared to influence the work reported in this paper.

## References

[1] Matthews J.R., Brutico J.M., Abraham D.T. (2022). Differences in clinical and functional outcomes between osteochondral allograft transplantation and autologous chondrocyte implantation for the treatment of focal articular cartilage defects. Orthop J Sports Med.

[2] Stannard J.P., Stannard J.T., Schreiner A.J. (2020). Fresh osteochondral allograft transplants in the knee: Bipolar and beyond. J Knee Surg.

[3] Merkely G., Ogura T., Ackermann J., Barbieri Mestriner A., Gomoll A.H. (2021). Clinical outcomes after revision of autologous chondrocyte implantation to osteochondral allograft transplantation for large chondral defects: A comparative matched-group analysis. Cartilage.

[4] Defroda S.F., Bokshan S.L., Yang D.S., Daniels A.H., Owens B.D. (2021). Trends in the surgical treatment of articular cartilage lesions in the United States from 2007 to 2016. J Knee Surg.

[5] Gilat R., Haunschild E.D., Huddleston H.P. (2021). Osteochondral allograft transplant for focal cartilage defects of the femoral condyles: Clinically significant outcomes, failures, and survival at a minimum 5-year follow-up. Am J Sports Med.

[6] Wang X., Ren Z., Liu Y. (2023). Characteristics and clinical outcomes after osteochondral allograft transplantation for treating articular cartilage defects: Systematic review and single-arm meta-analysis of studies from 2001 to 2020. Orthop J Sports Med.

[7] Migliorini F., Maffulli N., Baroncini A. (2022). Allograft versus autograft osteochondral transplant for chondral defects of the talus: Systematic review and meta-analysis. Am J Sports Med.

[8] Poursalehian M., Ghaderpanah R., Bagheri N., Mortazavi S.M.J. (2024). Osteochondral allografts for the treatment of shoulder instability: A systematic review and meta-analysis. Bone Jt Open.

[9] Ramkumar P.N., Karnuta J.M., Haeberle H.S., Rodeo S.A., Nwachukwu B.U., Williams R.J. (2021). Effect of preoperative imaging and patient factors on clinically meaningful outcomes and quality of life after osteochondral allograft transplantation: A machine learning analysis of cartilage defects of the knee. Am J Sports Med.

[10] Merkely G., Farina E.M., Leite C.B.G. (2022). Association of sex mismatch between donor and recipient with graft survivorship at 5 years after osteochondral allograft transplantation. Am J Sports Med.

[11] Kunze K.N., Ramkumar P.N., Manzi J.E., Wright-Chisem J., Nwachukwu B.U., Williams R.J. (2023). Risk factors for failure after osteochondral allograft transplantation of the knee: A systematic review and exploratory meta-analysis. Am J Sports Med.

[12] Wang D., Coxe F.R., Balazs G.C. (2018). Graft-recipient anteroposterior mismatch does not affect the midterm clinical outcomes of osteochondral allograft transplantation of the femoral condyle. Am J Sports Med.

[13] Gaul F., Tírico L.E.P., McCauley J.C., Bugbee W.D. (2018). Long-term follow-up of revision osteochondral allograft transplantation of the ankle. Foot Ankle Int.

[14] Bombard Y., Baker G.R., Orlando E. (2018). Engaging patients to improve quality of care: A systematic review. Implement Sci.

[15] Springer B., Dreisbach R., Schatz K.D., Kubista B., Waldstein W. (2023). Online videos regarding relevant postoperative patient information and postoperative rehabilitation after arthroscopic rotator cuff repair provide poor information quality, accuracy, and reliability. Arthroscopy.

[16] D’Ambrosi R., Milinkovic D.D., Abermann E., Herbort M., Fink C. (2024). Quality of YouTube videos regarding anterior cruciate ligament reconstruction using quadriceps tendon autograft is unsatisfactory. Arthroscopy.

[17] Springer B., Bechler U., Koller U., Windhager R., Waldstein W. (2020). Online videos provide poor information quality, reliability, and accuracy regarding rehabilitation and return to sport after anterior cruciate ligament reconstruction. Arthroscopy.

[18] Yüce A., İğde N., Ergün T., Mısır A. (2022). YouTube provides insufficient information on patellofemoral instability. Acta Orthop Traumatol Turc.

[19] Murray E., Lo B., Pollack L. (2003). The impact of health information on the internet on the physician-patient relationship: Patient perceptions. Arch Intern Med.

[20] Cole W.W., Perez-Chaumont A., Miskimin C., Mulcahey M.K. (2021). Social media and its use in orthopaedic surgery resident education and training. JBJS Rev.

[21] Jansen B.J., Spink A. An analysis of web documents retrieved and viewed. International Conference on Internet Computing. 2003. https://faculty.ist.psu.edu/jjansen/academic/pubs/pages_viewed.pdf.

[22] Bernard A., Langille M., Hughes S. (2007). A systematic review of patient inflammatory bowel disease information resources on the World Wide Web. Am J Gastroenterol.

[23] Mack J. (1997). Quality of medical information on the Internet. JAMA.

[24] Charnock D., Shepperd S., Needham G., Gann R. (1999). DISCERN: An instrument for judging the quality of written consumer health information on treatment choices. J Epidemiol Community Health.

[25] Wang D., Jayakar R.G., Leong N.L., Leathers M.P., Williams R.J., Jones K.J. (2017). Evaluation of the quality, accuracy, and readability of online patient resources for the management of articular cartilage defects. Cartilage.

[26] Sherman S.L., Garrity J., Bauer K., Cook J., Stannard J., Bugbee W. (2014). Fresh osteochondral allograft transplantation for the knee: Current concepts. J Am Acad Orthop Surg.

[27] Khan S.A., Baghdadi S., Carey J.L., Moores T.S., Sheth N.P., Ganley T. (2021). Osteochondral fractures after patellar dislocation: Current concepts. J Am Acad Orthop Surg Glob Res Rev.

[28] Gross C.E., Adams S.B., Easley M.E., Nunley J.A. (2016). Role of fresh osteochondral allografts for large talar osteochondral lesions. J Am Acad Orthop Surg.

[29] Doğan N.Ö. (2018). Bland-Altman analysis: A paradigm to understand correlation and agreement. Turk J Emerg Med.

[30] Schmidt S., Darwich A., Leutheuser S., Krahl D., Navas L. (2024). The use of social media in orthopedic and trauma surgery education: A cross-sectional survey of German-speaking residents and medical students. Healthcare (Basel).

[31] Madathil K.C., Rivera-Rodriguez A.J., Greenstein J.S., Gramopadhye A.K. (2015). Healthcare information on YouTube: A systematic review. Health Informatics J.

[32] Ghersi A., Mansour J., Marchand P., Al Rubaie A., Kouyoumdjian P., Coulomb R. (2022). Surgical videos on the internet: Is this a reliable pedagogical tool in residency training?. SICOT J.

[33] Kunze K.N., Cohn M.R., Wakefield C. (2019). YouTube as a source of information about the posterior cruciate ligament: A content-quality and reliability analysis. Arthrosc Sports Med Rehabil.

